# Ubiquitin-Like Proteasome System Represents a Eukaryotic-Like Pathway for Targeted Proteolysis in Archaea

**DOI:** 10.1128/mBio.00379-16

**Published:** 2016-05-17

**Authors:** Xian Fu, Rui Liu, Iona Sanchez, Cecilia Silva-Sanchez, Nathaniel L. Hepowit, Shiyun Cao, Sixue Chen, Julie Maupin-Furlow

**Affiliations:** a Department of Microbiology and Cell Science, Institute of Food and Agricultural Sciences, University of Florida, Gainesville, Florida, USA; b Proteomics and Mass Spectrometry, Interdisciplinary Center for Biotechnology Research, University of Florida, Gainesville, Florida, USA; c Department of Biology, College of Liberal Arts and Sciences, University of Florida, Gainesville, Florida, USA; d Genetics Institute, University of Florida, Gainesville, Florida, USA; e College of Food Science and Technology, Zhongkai University of Agriculture and Engineering, Guangzhou, Guangdong, China

## Abstract

The molecular mechanisms of targeted proteolysis in archaea are poorly understood, yet they may have deep evolutionary roots shared with the ubiquitin-proteasome system of eukaryotic cells. Here, we demonstrate in archaea that TBP2, a TATA-binding protein (TBP) modified by ubiquitin-like isopeptide bonds, is phosphorylated and targeted for degradation by proteasomes. Rapid turnover of TBP2 required the functions of UbaA (the E1/MoeB/ThiF homolog of archaea), AAA ATPases (Cdc48/p97 and Rpt types), a type 2 JAB1/MPN/MOV34 metalloenzyme (JAMM/MPN+) homolog (JAMM2), and 20S proteasomes. The ubiquitin-like protein modifier small archaeal modifier protein 2 (SAMP2) stimulated the degradation of TBP2, but SAMP2 itself was not degraded. Analysis of the TBP2 fractions that were not modified by ubiquitin-like linkages revealed that TBP2 had multiple N termini, including Met1-Ser2, Ser2, and Met1-Ser2(p) [where (p) represents phosphorylation]. The evidence suggested that the Met1-Ser2(p) form accumulated in cells that were unable to degrade TBP2. We propose a model in archaea in which the attachment of ubiquitin-like tags can target proteins for degradation by proteasomes and be controlled by N-terminal degrons. In support of a proteolytic mechanism that is energy dependent and recycles the ubiquitin-like protein tags, we find that a network of AAA ATPases and a JAMM/MPN+ metalloprotease are required, in addition to 20S proteasomes, for controlled intracellular proteolysis.

## IMPORTANCE

This study advances the fundamental knowledge of signal-guided proteolysis in archaea and sheds light on components that are related to the ubiquitin-proteasome system of eukaryotes. In archaea, the ubiquitin-like proteasome system is found to require function of an E1/MoeB/ThiF homolog, a type 2 JAMM/MPN+ metalloprotease, and a network of AAA ATPases for the targeted destruction of proteins. We provide evidence that the attachment of the ubiquitin-like protein is controlled by an N-terminal degron and stimulates proteasome-mediated proteolysis.

## INTRODUCTION

Highly selective and targeted protein turnover plays a pivotal role in intracellular homeostasis, signaling, transcription regulation, and protein quality control (1–3). In eukaryotes, the specificity and selectivity of proteolysis is mainly conferred by ubiquitylation, a posttranslational modification by which ubiquitin (Ub) is covalently attached to target proteins by E1-, E2-, and E3-type enzymes (4). Selective protein turnover often requires degradation signals, or “degrons,” in protein substrates. Degrons of protein substrates include biosynthetic errors or misfolding (5), specific amino acid regions (6), reversible posttranslational modifications like phosphorylation (7), cotranslational modifications like N^α^-acetylation (8), and N-terminal-residue identity (9, 10). In eukaryotes, degrons are frequently recognized by the ubiquitylation system, resulting in the formation of poly-Ub chains which serve as the signal for proteolysis by 26S proteasomes.

The 26S proteasomes of eukaryotes are multicatalytic, energy-dependent proteases that process substrates of the Ub-proteasome system (UPS) for cellular proteostasis. These enzymes are nanocompartmentalized and consist of 19S regulatory particles (RPs) and 20S catalytic core particles (CPs) which harbor the proteolytic active sites (11). CPs are formed from four stacked heptameric rings of α- and β-subunits and are conserved in all three domains of life (12). Protein degradation by proteasomes is proposed to be initiated by unstructured regions on substrates (13). Efficient protein degradation by CPs is assisted by AAA+ ATPases, including the RP Rpt1 to -6 subunits of eukaryotes and the related archaeal proteasome-activating nucleotidases (PANs), which unfold protein substrates, stimulate the opening of CPs, and translocate unfolded substrates into the CP channel for proteolysis (14–17). One key feature of UPS-mediated degradation is the removal of the poly-Ub on protein substrates by a deubiquitylating enzyme (DUB) (the RP Rpn11 subunit), prior to or coincident with translocation of unfolded substrates (18–20).

Homologs related to UPS are widely distributed across all archaeal genomes, suggesting the existence of an ancient pathway for targeted proteolysis in archaea. Ub-like proteins called SAMPs (small archaeal modifier proteins) were discovered in archaea by study of *Haloferax volcanii* (21, 22). SAMPs are isopeptides that are linked to lysine residues of protein substrates by a mechanism termed sampylation, which requires the noncanonical E1 enzyme, UbaA (21, 23, 24). Desampylation, the reverse of the process of sampylation, is achieved by DUB homologs of the JAB1/MPN/MOV34 metalloenzyme (JAMM/MPN+) subfamily, such as JAMM1 of *H. volcanii* (HvJAMM1) (25, 26). Although evidence reveals that sampylation is linked to archaeal proteasome function (21, 22, 27), little is known about the mechanism by which substrates are targeted for turnover by the SAMP-proteasome system (SPS; an analog of UPS), the components of SPS, or the degrons associated with the targeted proteolysis.

Here, we advance the fundamental knowledge of targeted proteolysis in archaeal cell biology by studying the SPS-mediated turnover of TATA-binding protein 2 (TBP2) through a mechanism apparently associated with N-terminal phosphorylation. We demonstrate that TBP2 is targeted for degradation by SPS, with efficient turnover requiring UPS homologs E1-like UbaA, the JAMM/MPN+-related HvJAMM2, and the AAA ATPases Rpt-like PAN2 and Cdc48c. We provide evidence that SPS-mediated turnover of TBP2 is stimulated by phosphorylation of Ser2, which occurs without the removal of N-terminal Met1. Overall, this work reveals that proteins can be targeted for controlled proteolysis by the SPS in archaea, thus deepening our understanding regarding the origins of the eukaryotic UPS.

## RESULTS

### Abundance of TBP2 is increased by mutation of the SAMP-proteasome system.

TBP2 is one of four TATA-binding protein paralogs in *Haloferax volcanii* and is found isopeptide-linked at lysine residues to the C terminus of Ub-like SAMP2 (21). By analogy to Ub tagging in eukaryotes, we hypothesized that sampylation in archaea serves as a signal for targeting proteins such as TBP2 for destruction by proteasomes or, alternatively, is a nonproteolytic mechanism to control TBP2’s association with protein partners.

**FIG 1:**
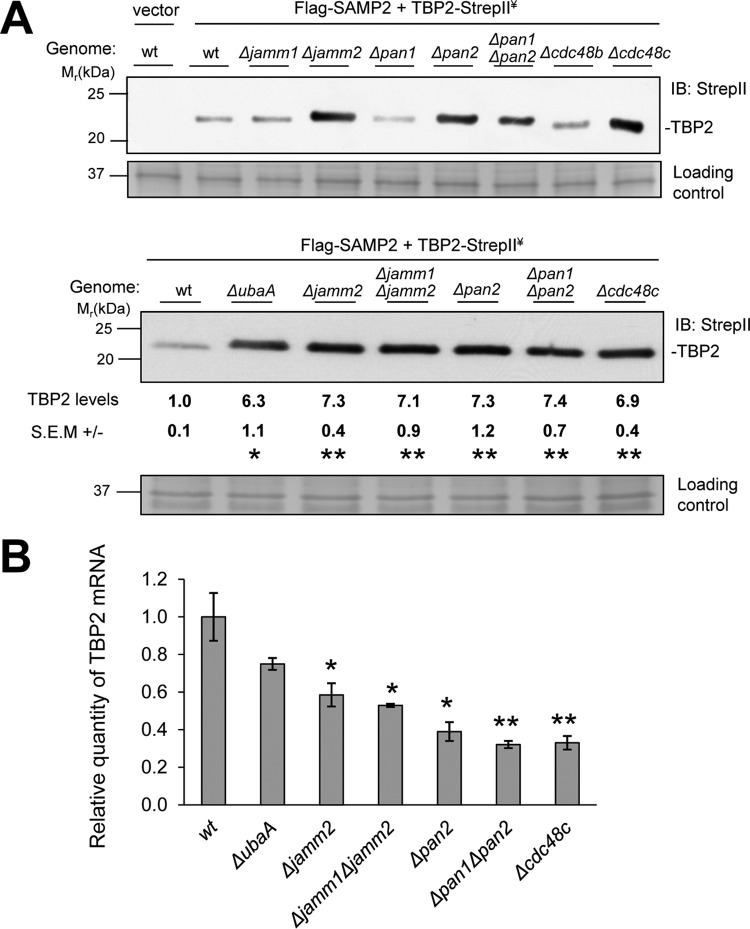
The abundance of TBP2 is increased by mutation of the SAMP-proteasome system. (A) TBP2 protein levels in wild-type (wt) and SPS deletion strains determined by anti-StrepII antibody immunoblotting (IB). TBP2 was fused to a C-terminal StrepII tag, coexpressed with Flag-SAMP2, and monitored by anti-StrepII antibody IB. Equal loading was confirmed by Coomassie blue (CB) staining. Relative levels of TBP2 protein in SPS deletion strains versus the level in the wt are indicated below the gels. Data were quantified by ImageJ. Data represent mean results ± standard errors of the means (SEM) from three independent experiments (*, *P* < 0.01; **, *P* < 0.001). ¥, expressed in *trans*. (B) Histogram showing relative TBP2-StrepII transcript levels as determined by qRT-PCR. The mRNA levels were normalized to the level of the internal standard *ribL*. Data represent mean results ± SEM from three independent experiments (*, *P* < 0.05; **, *P* < 0.01). *P* values were determined by two-tailed, unpaired Student’s *t* test. See Materials and Methods for details.

To initiate this study, the steady-state levels of TBP2 were compared in wild-type (wt) and SPS mutant strains. TBP2 levels were found to be increased ~sevenfold upon deletion of the SPS genes, including *ubaA*, *jamm2*, *pan2*, and *cdc48c* (UbaA is the E1-like SAMP-activating enzyme, JAMM2 is a JAMM/MPN+ homolog, PAN2 is an Rpt-like AAA ATPase, and Cdc48c is a member of the Cdc48/p97 AAA ATPase family) (Fig. 1A). In contrast, deletion of *jamm1* (encoding the desampylase HvJAMM1) and the AAA ATPase *pan1* and *cdc48b* genes had little if any influence on the abundance of TBP2 (Fig. 1A). Quantitative reverse transcriptase PCR (qRT-PCR) analysis revealed that the increased levels of TBP2 protein in the SPS mutant strains were not due to differences in transcript levels, as the transcripts specific for TBP2 were less abundant in SPS mutants than in the wt (Fig. 1B). Thus, mutation of specific genes within the SPS caused a posttranscriptional increase in TBP2 protein levels and may have triggered an autoregulatory response that reduced TBP2 transcript levels. Autoregulation by transcriptional repression of transcription factors is common (28) and feasible, as TBP2 was expressed (tagged with Strep-tag II [hereinafter referred to as StrepII]) from a promoter with a TATA box consensus sequence known to bind TBPs.

### Abundance of TBP2 is increased by chemical inhibition of 20S proteasomes and Cdc48/p97-type ATPases.

**FIG 2:**
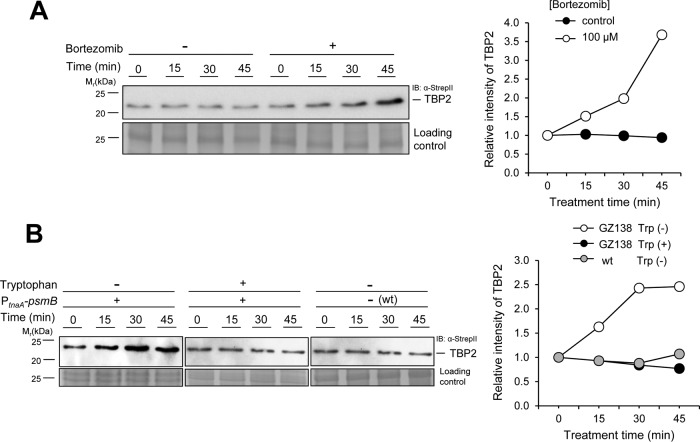
The abundance of TBP2 is increased by inhibition of 20S proteasomes. (A) Abundance of TBP2 in wild-type (wt) cells treated with an inhibitor of proteasomal CPs (bortezomib, +) or a mock control (dimethylformamide [DMF], −). (B) Right, TBP2 abundance in GZ138 (P*_tnaA_*-*psmB*) cells grown in medium containing or lacking tryptophan, as well as wt cells grown in medium lacking tryptophan, for the indicated times. Quantification of relative TBP2 protein levels is expressed as fold change from time zero, which was set at 1.0. Left, TBP2-StrepII was coexpressed with Flag-SAMP2 and monitored by anti-StrepII antibody IB. Equal loading was confirmed by CB staining. Experiments were performed in at least biological duplicates, and representative images are shown. Data were quantified by ImageJ. See Materials and Methods for details.

We next examined whether the levels of TBP2 protein could be stabilized in an archaeon by the addition of UPS inhibitors. TBP2 levels were analyzed in *H. volcanii* cells treated with bortezomib (PS-341; Velcade), which reversibly binds and inhibits the catalytic site of proteasomal CPs with high affinity and specificity (29) (50% inhibitory concentration [IC_50_] of <40 nM for *H. volcanii* CPs; McMillian and J. Maupin-Furlow, unpublished results). The level of TBP2 protein was found to be increased by ~3.5-fold compared to its level in the mock control after a 45-min treatment with bortezomib (Fig. 2A). Likewise, treatment of *H. volcanii* cells with *N*^2^,*N*^4^-dibenzylquinazoline-2,4-diamine (DBeQ), a selective and potent inhibitor of the highly conserved p97/Cdc48 ATPases (30), increased the abundance of TBP2 by ~twofold compared with its level in the mock control (see Fig. S1 in the supplemental material). These results reveal that treatment of archaeal cells with UPS inhibitors can increase the abundance of proteins, such as TBP2, which are tagged by Ub-like bonds.

### Abundance of TBP2 is increased by depletion of 20S proteasomes.

We next studied whether the abundance of TBP2 could be increased by genetic depletion of proteasomal CPs. To test this idea, TBP2 levels were analyzed in GZ138, a strain with an insertion of the tryptophan-dependent tryptophanase promoter (P_tnaA_) upstream from *psmB*, encoding the CP β subunit which is essential in *H. volcanii* (31). Cells were cultured on medium with and without tryptophan, the inducer of P*_tnaA_*-*psmB* gene expression. TBP2 levels were found to be notably increased within 45 min after P*_tnaA_*-*psmB* gene expression was repressed by the removal of tryptophan from the culture medium (Fig. 2B). In contrast, the steady-state levels of TBP2 remained constant when the medium was supplemented with tryptophan (Fig. 2B). As an added control, tryptophan was removed from wt cells, in which *psmB* was regulated by its native promoter, and the levels of TBP2 were found to be unaltered (Fig. 2B). Therefore, the abundance of TBP2 was increased by genetic depletion of the CPs, thus providing evidence that TBP2 is targeted for degradation by proteasomes.

### TBP2 is stabilized by mutation of the SAMP-proteasome system.

**FIG 3:**
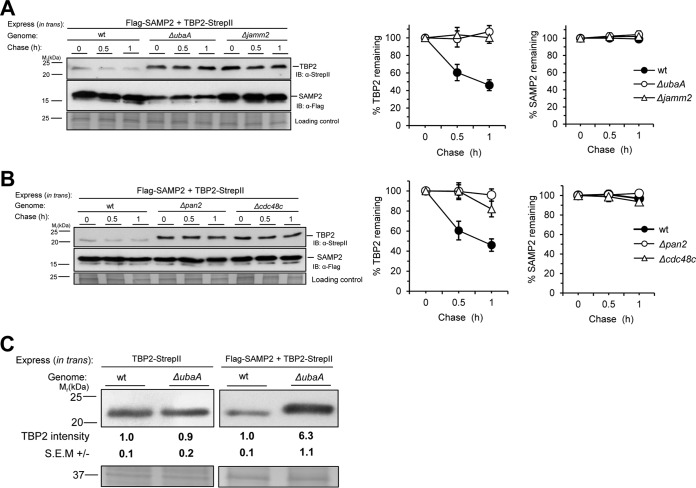
TBP2 is stabilized by mutation of the SAMP-proteasome system. (A and B) Left, chase assays were performed in wild-type (wt) and SPS gene deletion strains expressing Flag-SAMP2 and TBP2-StrepII. Log-phase cells were treated with 20 µg·ml^−1^ actinomycin D and 50 µg·ml^−1^ anisomycin for the indicated times and collected. TBP2 and SAMP2 protein levels were determined by anti-StrepII and anti-Flag antibody IB, respectively. Equal loading was confirmed by CB staining. Right, quantification of TBP2 and SAMP2 intensities in chase assays. Results are expressed as the percent change from time zero, which was set at 100%. Data represent the mean results ± SEM from three independent experiments. (C) TBP2 protein levels in wild-type (wt) and *ΔubaA* strains determined by anti-StrepII antibody immunoblotting. TBP2-StrepII was expressed alone (left) and coexpressed with Flag-SAMP2 (right). Relative levels of TBP2 protein in the *ΔubaA* strain versus the wt are indicated below. Equal loading was confirmed by Coomassie blue (CB) staining. Experiments were performed in triplicate, and representative images are shown. Data were quantified by ImageJ. See Materials and Methods for details.

In order to determine whether the accumulation of TBP2 protein that was observed in the SPS mutants was due to an increase in the rate of translation or a decrease in the rate of protein turnover, a chase assay was performed. Cells were treated with inhibitors of translation (anisomycin) and transcription (actinomycin D) to minimize the synthesis of new protein, and TBP2 protein levels were monitored by immunoblotting (IB) and densitometric analysis. During the 1-h chase period, TBP2 was found to be degraded in wt cells, whereas little if any TBP2 turnover was observed in the *ΔubaA* and *Δjamm2* strains and the half-life of TBP2 was increased 7- to 16-fold in the *Δpan2* and *Δ*c*dc48c* strains (Fig. 3A and B; see also Fig. S2 in the supplemental material). Optimal levels of SAMP2 were required to target TBP2 for SPS-mediated turnover, based on a finding that the ratio of the amount of TBP2 protein in the wt to the amount in the *ΔubaA* mutant strain was decreased when *tbp2* was ectopically coexpressed with *samp2* compared with the ratio found with the expression of *tbp2* alone (Fig. 3C). Together, these results demonstrate that a major transcription factor of archaeal cells, TBP2, is degraded in an SPS-dependent manner.

### SAMP2 is not targeted for degradation by archaeal proteasomes.

The Ub-like SAMP2 is correlated with TBP2 degradation but is itself not degraded. Further analysis of the SAMP2 degradation rate in the chase assay revealed that SAMP2 was highly stable. Although TBP2 was clearly degraded in wt cells, SAMP2 turnover was not detected in either wt or SPS mutant strains (Fig. 3A and B; see also Fig. S2 in the supplemental material). Anjum et al. proposed as a model that SAMPs are degraded together with their substrates by 20S CP proteasomes in archaea (27). This model was based on *in vitro* degradation of a linear fusion of a SAMP homolog to green fluorescent protein (GFP) (27) and by analogy to the disordered Pup (prokaryotic Ub-like protein) tag of mycobacteria that is codegraded with its protein targets by proteasomes assisted by ATPases (17). Here, we provide evidence that the Ub-like SAMP2 is not targeted for degradation by the archaeal SPS and is instead recycled by the DUB-like protease, JAMM2. The Rpn11 JAMM/MPN+ metalloprotease homolog, JAMM2, is our proposed candidate for recycling SAMP2, as the deletion of *jamm2* stabilizes and increases the abundance of TBP2 in the cell.

### Detection of TBP2 conjugates modified by SAMPs.

**FIG 4:**
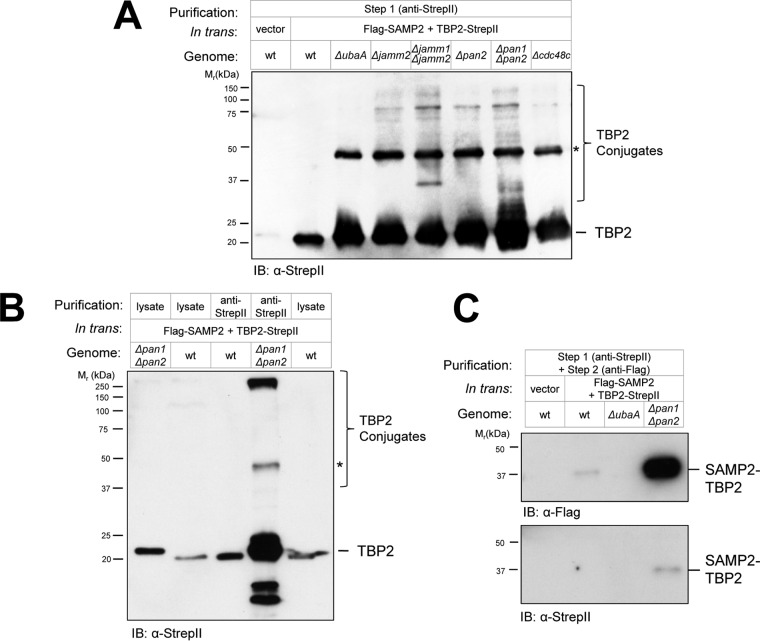
Detection of TBP2 conjugates modified by SAMPs. TBP2 was enriched from wild-type (wt) and SPS deletion strains by Strep-Tactin (anti-StrepII antibody) (A and B) and tandem affinity purification (C), which included a subsequent immunoprecipitation by anti-Flag antibody. TBP2-StrepII was ectopically expressed with Flag-SAMP2 in the wt and SPS mutant strains and compared to an empty-vector control. Equivalent amounts of cell lysates, as determined by BCA assay, were applied to the Strep-Tactin resin. TBP2, TBP2 conjugates (including the Ubl-modified form, SAMP2-TBP2), and an anti-StrepII antibody signal likely to be a TBP2 dimer (*) are indicated. See Materials and Methods for details.

If sampylation preceded proteasome-mediated turnover of TBP2, we hypothesized that this transcription factor would accumulate in ubiquitin-like (Ubl) protein-modified forms in SPS mutants that were dysfunctional in proteasome function but not sampylation. To test this hypothesis, TBP2 was enriched by anti-StrepII antibody affinity chromatography from wt and SPS mutant strains and analyzed by reducing SDS-PAGE. By this approach, TBP2 was found to be more abundant and to be in various high-molecular-mass forms in the SPS mutants (*ΔubaA*, *Δjamm Δjamm2*, *Δjamm2*, Δ*pan1* Δ*pan2*, *Δpan2*, and *Δcdc48c* strains) compared with its occurrence in the wt (Fig. 4A and B). Of particular note was a 48-kDa form of TBP2 that was absent in the wt and yet abundant in all of the SPS mutants examined (Fig. 4A and B). We speculate that this form of TBP2 is a dimer that forms when TBP2 is at high concentration, since this form was detected in the E1-like mutant strain (*ΔubaA* strain), which does not synthesize Ubl bonds (22, 24). Other high-molecular-mass forms of TBP2 were observed in the *Δjamm Δjamm2* and Δ*pan1* Δ*pan2* strains that were not detected in the *ΔubaA* mutant or wt cells (Fig. 4A and B). Often, these forms of TBP2 were found to be recalcitrant to separation by reducing SDS-PAGE and to be retained in the stacking gel (Fig. 4B, the >250-kDa band, for example) similarly to Ub-modified proteins (e.g., see reference 32). Low-molecular-mass fragments that were potentially degradation products of TBP2 were also uniquely detected in Δ*pan1* Δ*pan2* mutants and not in wt cells (Fig. 4B). These results suggested that sampylated forms of TBP2 were increased in abundance by SPS mutations, including proteasomal ATPase (Δ*pan1* Δ*pan2*) and desampylase (*Δjamm Δjamm2*) gene deletions.

To further understand the covalently modified forms of TBP2 that accumulated in the SPS mutants with dysfunctional proteasomal machineries, the Ubl-SAMP2-modified proteins were isolated from the TBP2-StrepII enrichment fractions by Flag-SAMP2 immunoprecipitation. A SAMP2-TBP2 conjugate(s) of 37 kDa was found to be readily purified by this approach. Compared with its level in the wt, the SAMP2-TBP2 conjugate was found to be more abundant in the Δ*pan1* Δ*pan2* mutant and was not detected in the *ΔubaA* control (Fig. 4B). The ~17-kDa increase in the migration of TBP2 due to the SAMP2 linkage suggested that TBP2 was di- or monosampylated. In contrast, the TBP2 species that were identified at >50 kDa in the TBP2-StrepII enrichment fractions of the SPS mutants but not in the *ΔubaA* and wt strains (Fig. 4A and B) were not enriched by this approach (Fig. 4C). We speculate that the polysampylated forms of TBP2 were not stable or amenable to the tandem affinity purification approach. Alternatively, the N-terminally Flag-tagged SAMP2 was only a minor portion of the polymeric SAMP chains, and instead, SAMP moieties encoded by the genomic copies of *samp1* to *-3* were attached to TBP2 in these chains. Interestingly, in *Mycobacterium tuberculosis*, at least one substrate of the Pup proteasome system migrates as multiple covalent forms by SDS-PAGE, including large-molecular-mass species that are not detected as pupylated by anti-Pup antibody IB (33). The low yield of TBP2-SAMP2 conjugates purified from the wt compared with the yield from the Δ*pan1* Δ*pan2* mutant is consistent with a model that Ub-like SAMP2 modification serves as a signal for targeting proteins such as TBP2 for destruction by energy-dependent PAN-proteasomes.

### Two apparent forms of “unsampylated” TBP2.

**FIG 5:**
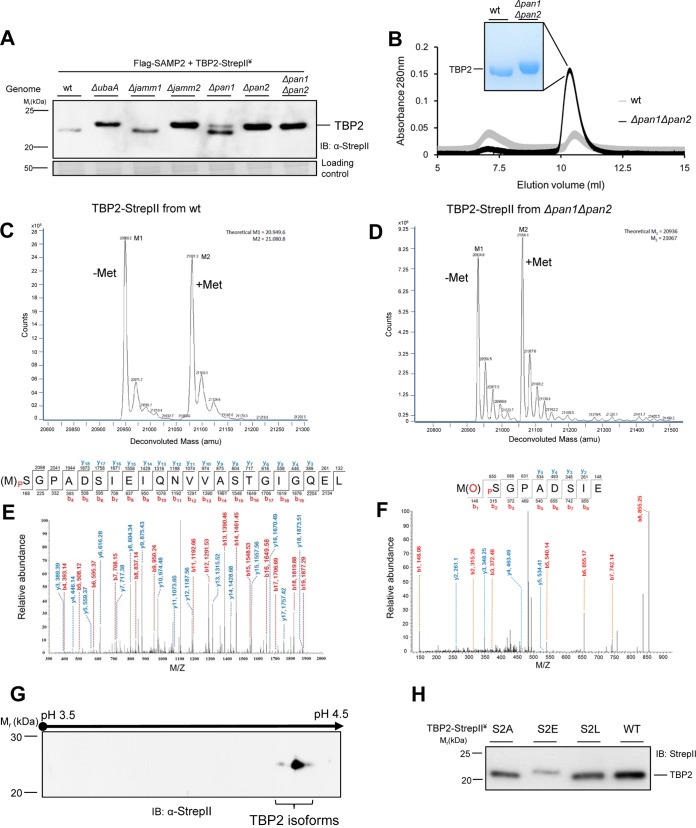
Characterization of TBP2 modification linked with SPS. (A) Mobility shifts of TBP2 in SPS deletion strains compared to the mobility of the wt. TBP2 was detected by anti-StrepII antibody IB of cell lysate separated by reducing 14% SDS-PAGE. (B) TBP2 samples purified from wt and SPS mutant (Δ*pan1* Δ*pan2*) strains by StrepII affinity chromatography and gel filtration chromatography (e.g., 9 and 78 µg of protein purified per liter of culture from wt and Δ*pan1* Δ*pan2* mutant cells, respectively). TBP2 fractions that eluted at ~20 to 25 kDa by gel filtration chromatography were separated (1 µg per lane) by reducing 12% SDS-PAGE and stained with CB. (C and D) ESI-MS analysis of TBP2 purified from wt and Δ*pan1* Δ*pan2* strains, as indicated. M1 (Met1-cleaved TBP2) and M2 (intact TBP2) represent the observed and theoretical average masses in panel C and the observed and theoretical monoisotopic masses in panel D. (E and F) Representative tandem mass spectrometry (MS/MS) spectra of N-terminal peptides derived by collision-induced dissociation of the doubly charged precursor after chymotrypsin (E) and Glu-C and trypsin (F) digestion of TBP2. High mass accuracy MS/MS unambiguously confirmed Ser2 phosphorylation of TBP2 based on the near complete matches of *b*- (colored red) and *y*-type ions (colored blue). (G) Anti-StrepII antibody IB of TBP2 isoforms separated by 2-D gel electrophoresis from the Δ*pan1* Δ*pan2* mutant. (H) The mobility shift of TBP2 wt and variant (S2A, S2E, and S2L) proteins expressed in wt cells and detected by anti-StrepII antibody IB of cell lysates separated by reducing 14% SDS-PAGE. (A to H) ¥, all strains coexpressed TBP2-StrepII with Flag-SAMP2 in *trans*. See Materials and Methods for details.

By optimizing the conditions of reducing SDS-PAGE to separate proteins in the 20- to 25-kDa range, we found unsampylated TBP2 to be shifted to a more slowly migrating form when its abundance was increased by SPS mutation. Compared with the wt, the SPS mutations *ΔubaA*, *Δjamm2*, and *Δpan2* were found to slow the migration of TBP2 in these gels (Fig. 5A). The *Δpan1* mutation had a partial effect on TBP2 migration (Fig. 5A), which may have been due to perturbation of the PAN1 and -2 heterooligomers that form in *H. volcanii* cells (34, 35). We suspected that an increase in acidity, such as by phosphorylation, could account for the observed slowly migrating form of TBP2. Phosphorylation can retard the SDS-PAGE migration of proteins with a high content of acidic residues near the site of posttranslational modification (36) and can regulate the ubiquitylation and degradation of proteins by UPS in eukaryotic cells (6, 37). The retarded migration of the unsampylated form of TBP2 in the SPS mutants suggested that TBP2 was subjected to an additional type of co-/posttranslational modification that was distinct from sampylation and that was correlated with the regulated SPS-mediated turnover of TBP2.

### TBP2 is a mixture of N-terminal Met1-removed and intact forms.

In order to determine the type of co-/posttranslational modification that could precede and regulate sampylation, we investigated the composition of unsampylated TBP2 by mass spectrometry (MS). This form of TBP2 was reproducibly purified by affinity chromatography and gel filtration to 9- to 10-fold higher yields from the Δ*pan1* Δ*pan2* mutant than from the wt (Fig. 5B). These results are consistent with our finding that TBP2 levels were much higher in the cell lysate of the Δ*pan1* Δ*pan2* mutant than in that of the wt (Fig. 1B). For initial investigation of the posttranslational modification that appeared to precede sampylation of TBP2, the accurate mass of the purified TBP2 was determined at high resolution by direct-infusion electrospray ionization-time of flight (ESI-TOF) mass spectrometry analysis. Two isoforms of TBP2 were detected by this approach, including Met1-removed and intact forms (Fig. 5C and D). The Met1-removed form was presumed to be cleaved by the endogenous methionine aminopeptidase (MetAP), as MetAPs specifically remove N-terminal Met1 if the amino acid residue at the second position is nonbulky and uncharged (e.g., Gly, Ala, Ser, Cys, Pro, Thr, and Val) (38). The deduced N terminus of TBP2 is Met1-Ser2, with Ser2 likely promoting the removal of Met1 by MetAP. Based on shotgun MS analysis of haloarchaeal proteomes, proteins with N-terminal Met1-Ser2 are commonly detected as a mixture of Met1-cleaved and intact forms (39, 61–62). Thus, our finding that TBP2 was in Met1-removed and intact forms was not unique to this transcription factor. In fact, the mixture of these two forms of TBP2 in the wt was at a ratio similar to that in the Δ*pan1* Δ*pan2* mutant (Fig. 5C and D) and, thus, could not be attributed to the observed differences in TBP2 levels between these strains. In line with this finding, the removal of Met1 would not alter the protein charge and would result in a difference in molecular mass (131 Da) not readily detected by reducing SDS-PAGE. Together, these results reveal TBP2 to be in Met1-removed and -intact forms; however, this posttranslational modification is not the distinguishing feature of the two TBP2 isoforms that are altered in abundance by SPS mutation and observed by SDS-PAGE.

### Ser2 of TBP2 is phosphorylated.

Our detection of phosphorylation using an ESI-TOF MS approach was likely hindered by the selective suppression of the ionization of phosphoproteins in the presence of their unmodified state (40); thus, we further characterized the modification(s) of TBP2 by reverse-phase liquid chromatography–tandem mass spectrometry (LC-MS/MS). Unsampylated TBP2 purified from the Δ*pan1* Δ*pan2* mutant was in-gel digested with chymotrypsin, and the phosphopeptides generated from this digestion were enriched by metal oxide affinity chromatography (MOAC). MS/MS analysis of these fractions revealed a nearly complete *y* and *b* ion series for a phosphopeptide that mapped to the N terminus of TBP2 with Met1 cleaved and Ser2 phosphorylated [Ser2(p)] (Fig. 5E). As chymotrypsin hydrolyzes amide bonds at low rates, particularly those with Met at the P1 position (41), the absence of Met1 in the Ser2(p)-containing peptide fragment could have been an artifact of the chymotrypsin treatment. Acidic residues at the P1′ position of the P1 ↓ P1′ bond are disfavored for cleavage by MetAPs (42); thus, phosphorylation of Ser2 would add a negative charge at the P1′ position that would prevent MetAP from removing Met1. In order to answer this next question, trypsin and Glu-C were utilized instead of chymotrypsin to generate appropriate N-terminal peptides for LC-MS/MS analysis. The only detection of a Ser2(p) form of TBP2 by the latter approach was when the Met1 was intact (Fig. 5F). In fact, with 94% coverage of TBP2 by LC-MS/MS analysis, Ser2 phosphorylation appeared to be the only posttranslational modification on the unsampylated TBP2, besides Met1 cleavage, and the latter did not occur in the Ser2 phosphorylated form. Consistent with the LC-MS/MS results, at least two isoforms of TBP2 (very likely phosphorylated and nonphosphorylated forms) with very similar molecular masses but distinct pIs were detected by anti-StrepII antibody immunoblotting of total proteins from the Δ*pan1* Δ*pan2* mutant separated by two-dimensional (2-D) gel electrophoresis (Fig. 5G). Thus, TBP2 was found to be modified by Ser2 phosphorylation, but only when Met1 was intact.

### TBP2’s migration and abundance are altered by the replacement of Ser2 with Glu.

In order to study whether phosphorylation was responsible for the SDS-PAGE migration differences of TBP2 observed in the wt and SPS mutants, a site-directed mutagenesis approach was used. TBP2 Ser2 was altered to (i) Ala (substitution denoted as S2A) to prevent phosphorylation, (ii) Glu (S2E) to mimic phosphorylation, and (iii) Leu (S2L) to prevent phosphorylation, as well as Met1 cleavage. Based on reducing SDS-PAGE analysis of these variant proteins, the S2A and S2L substitutions were found to have little if any impact on the migration of TBP2, while the S2E exchange shifted TBP2 to a more slowly migrating form (Fig. 5H) that was analogous to what was observed by SPS mutation (Fig. 5A). We next compared the steady-state levels of the TBP2 variant (S2A, S2E, and S2L) proteins in wt and SPS mutant strains. The S2A substitution was found to abolish the accumulation effect otherwise observed for TBP2 in the SPS mutant strains (see Fig. S3A in the supplemental material), suggesting that TBP2 S2A turnover was no longer regulated by SPS. Furthermore, the S2E exchange, which mimicked the phosphorylated-Ser2 form of TBP2, was found to dramatically decrease the level of TBP2 protein but not the transcript level in wt cells (see Fig. S3A and B). Consistent with our model, the levels of TBP2 S2E were found to be significantly higher (at a statistical significance of *P* < 0.05) in the *ΔubaA* and Δ*pan1* Δ*pan2* mutant strains than in the wt. However, the levels of these differences were modest, suggesting that the S2E phosphomimic, which is not susceptible to dephosphorylation, may be more prone to degradation by SPS-independent mechanisms than the S2(p) form. Sampylation may, therefore, be used to drive the proteolysis pathway forward and minimize reversal by phosphatases. Taken together, these data suggest that the phosphorylation of TBP2 Ser2 is linked to the stability of TBP2, which is regulated by SPS.

## DISCUSSION

**FIG 6:**
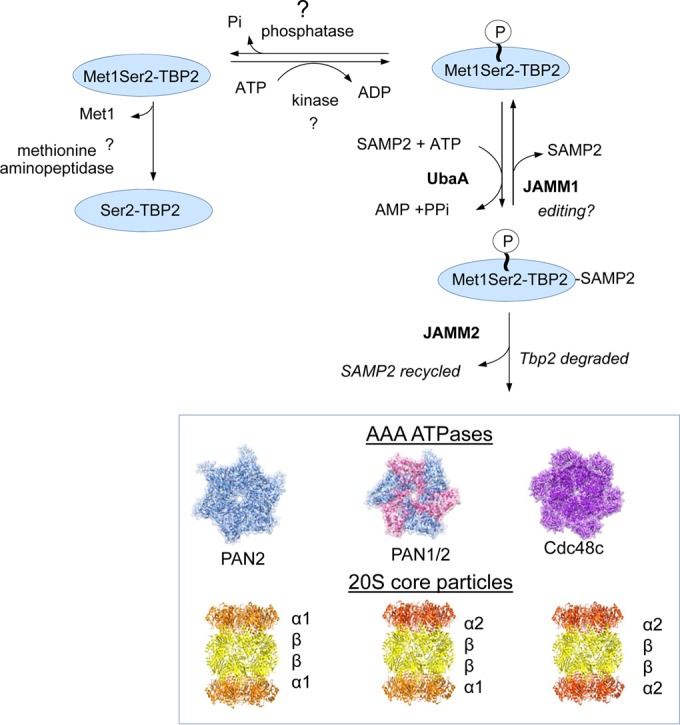
Model of regulated turnover of TBP2 by the archaeal SAMP proteasome system (SPS). In this model, the phosphorylation status of TBP2 Ser2 is an important factor that regulates TBP2 turnover by the archaeal SPS. The kinase and phosphatase enzymes that control phosphorylation of TBP2 Ser2 and the methionine aminopeptidase that cleaves TBP2 Met1 have yet to be identified. However, evidence suggests that the addition of a phosphoryl group to Ser2 inhibits the ability of methionine aminopeptidase to remove Met1 from TBP2. The Met1-Ser2(p) form of TBP2 is thought to be susceptible to sampylation by the E1-like UbaA and destruction by the archaeal SPS. The proteasomal AAA ATPases (Rpt-like PAN2 and Cdc48c) are used to recognize, unfold, and translocate TBP2 into the proteolytic chamber of the CPs for destruction by the proteasome system, while JAMM2 would remove and recycle the SAMP2 moiety from the TBP2 substrate during this process. JAMM1 is a desampylase that independently cleaves SAMPs from target proteins and, thus, may serve as a proofreading enzyme to ensure proper substrate recognition by the archaeal SPS.

Although the UPS has been known for decades to constitute the primary pathway for targeted proteolysis in eukaryotic cells, the biological roles of prokaryotic forms of ubiquitylation are less understood. In mycobacteria, Pup modification (pupylation) serves as a signal for proteasome-mediated degradation through a mechanism that is distinct from ubiquitylation (43). However, the archaeal system(s) responsible for targeted and selective proteolysis remain largely unclear. Here, we report the targeted proteolysis of TBP2 by the SPS in the archaeon *H. volcanii*. By integrating key findings from this study, we propose a working model of targeted protein turnover in archaea that is mediated by the SPS and regulated by the N-terminal state of the substrate protein (Fig. 6).

Unique to this study is our finding that JAMM2 can alter protein turnover in archaea. In this case, the deletion of *jamm2* caused a decrease in the turnover rate and an increase in the abundance of TBP2 in the cell, where TBP2 is a known target of sampylation by SAMP2. JAMM2 is a member of the JAMM/MPN+ metalloprotease family and is related to Rpn11 of the 26S proteasome. We propose that JAMM2 is used to recycle Ub-like SAMPs during SPS-mediated proteolysis. JAMM2 may be needed to promote efficient SPS-targeted proteolysis of TBP2 by removing SAMP2, which could otherwise sterically block the translocation of the substrate into the proteolytic chamber of proteasomal CPs. While our data suggest that archaea utilize a Ub-like recycling mechanism during SPS mediated proteolysis, Anjum et al. (27) have proposed a different model in which the SAMP tag stimulates substrate binding to the 20S CP proteasome; the SAMP is then codestroyed with the substrate by threading itself into the proteasome chamber. Consistent with our model, SAMP2 is highly stable compared with TBP2 when analyzed by chase assay, strongly suggesting that SAMP2 is recycled and not degraded along with its substrates *in vivo*. By analogy to Rpn11, which requires association with the ATP-hydrolyzing 19S lid of 26S proteasomes for its DUB activity (20), we speculate that JAMM2 needs to associate with protein partners to perform its desampylase activity. This speculation is consistent with the previous findings that *H. volcanii* JAMM2 (HvJAMM2) purified from recombinant *E. coli* is not functional (25), and the JAMM of *Archaeoglobus fulgidus*, which clusters in the same protein group as HvJAMM2, is also enzymatically inactive (44). In contrast, HvJAMM1 alone is found to cleave SAMP conjugates both *in vitro* and *in vivo* (22, 25, 26). Thus, it will be important to investigate the proteins associated with HvJAMM2 and determine whether or not streamlined forms of 19S RP-like complexes exist in archaeal proteasomes.

We expect that AAA ATPase modules (Cdc48 and Rpt-like PANs) are essential for SPS-mediated proteolysis in archaeal cells. AAA ATPases are likely important for recognizing Ub-like SAMP tags, in addition to their well-known role in the unfolding and translocation of substrate proteins into proteasomes (for a review, see reference 12). In particular, PAN2 appears to be the major Rpt-like AAA ATPase involved in TBP2 degradation, based on our finding that TBP2 and, especially, SAMP2-TPB2 accumulated notably in the *Δpan2* strain. It is interesting to note that *H. volcanii* PAN2 lacks the HbYX motif (where Hb is a hydrophobic residue, Y is tyrosine, and X is any amino acid) that can trigger conformational changes of CPs (16) and stimulate the degradation of substrates by proteasomes (45), while it has more extended N-terminal coiled-coil pairs than PAN1. SAMP1 is found to interact with an N-terminal peptide derived from PAN2 with weak affinity *in vitro* (46). Thus, N-terminal substrate recognition domains of the Rpt-like PAN2 may play an important role in recognizing SAMP2-modified TBP2 for degradation in archaea. Furthermore, this study provides the first *in vivo* evidence that a Cdc48-type AAA ATPase, which can interact with archaeal proteasomal CPs and stimulate the degradation of artificial substrates by CPs *in vitro* (47, 48), is linked to targeted proteolysis by archaeal proteasomes. TBP2 turnover was found to be impaired by genetic depletion of Cdc48c and chemical inhibition of p97/Cdc48 ATPases in *H. volcanii*. Accordingly, we propose that differential utilization of AAA ATPase modules (Cdc48 and Rpt-like PANs) by archaeal proteasomes serves as a strategy to enhance the capacity and functional flexibility of SPS-mediated proteolysis.

Degrons are important signals used to target proteins for destruction, and they likely function in archaea. In eukaryotes, degrons of UPS substrates include posttranslational modification by phosphorylation and sumoylation, as well as cotranslation modification by N^α^-acetylation (8, 49, 50). Compared to those of eukaryotes, the degrons of the archaeal SPS are not well characterized. Indirect evidence for archaeal degrons includes findings that phosphorylated proteins accumulate in a PAN1 mutant (*Δpan1* strain) (51) and that the replacement of the N-terminal penultimate residue of the α1 subunit of proteasomal CPs causes a notable accumulation of α1 protein in *H. volcanii* (52). In the work presented here, we found that TBP2 turnover by SPS is correlated with phosphorylation of Ser2 in TBP2, revealing that the archaeal SPS is elaborately regulated by additional types of modification for signal-guided and selective proteolysis. Interestingly, three of four TBPs in *H. volcanii* contain a permissive phosphorylation site (Ser or Thr) at the second position. We suspect that the archaeal SPS has a role in transcriptional regulation by modulating the abundance of specific TBPs in relationship to the levels of the other TBPs. In the haloarchaeon *Halobacterium salinarum*, the deletion of *serB*, encoding the only predicted phosphoserine phosphatase, revealed a striking increase of phosphorylated sites, including a large proportion of N-terminal Ser2 sites (53). Thus, it will be important to investigate whether or not the phosphorylation of the N-terminal penultimate residue is associated with SPS for other cellular proteins. Ub pathway degrons require specific E3 ligases (6). However, gene homologs of E2 and E3 in ubiquitylation are not conserved in the SAMP system. Further studies will be necessary to understand how the additional types of modification are recognized by the archaeal SPS for the regulation of targeted proteolysis.

## MATERIALS AND METHODS

### Strains and culture conditions.

The strains used in this study are summarized in Table S1 in the supplemental material. *Escherichia coli* TOP10 was used for recombinant DNA experiments. Plasmid DNAs used for transformation into *H. volcanii* were purified from *E. coli* strain GM2163. *E. coli* strains were grown aerobically in Luria-Bertani medium at 37°C. *H. volcanii* strains were grown at 42°C in ATCC 974 and glycerol minimal (GM) medium as previously described. Cells were grown aerobically in liquid medium with rotary shaking at 200 rpm or on plates (solid medium with 2% [wt/vol] agar). Ampicillin (0.1 mg·ml^−1^) and novobiocin (0.2 µg·ml^−1^) were added as needed for culturing *E. coli* and *H. volcanii* strains, respectively. Cells were stored at −80°C in 20% (vol/vol) glycerol stocks. *H. volcanii* cells from the freezer were routinely streaked onto ATCC 974 agar plates for isolation, and freshly isolated colonies were inoculated and grown to log phase in 4 ml ATCC 974 medium. To monitor the effect of bortezomib (LC Laboratories) on TBP2 abundance, log-phase cells were subcultured into 4.4 ml fresh ATCC 974 medium and grown to log phase (optical density at 600 nm [OD_600_] of 0.5 to 0.7). Bortezomib (2.2 µl of 200-mM bortezomib stock dissolved in dimethylformamide [DMF] at 99.8% [wt/vol]) and DMF solvent alone (2.2 µl DMF at 99.8% [wt/vol]) were added to log-phase cells (final concentrations of 100 µM bortezomib and 25 mM DMF). For regulation of the synthesis of proteasomal CPs, log-phase cells were subcultured into 4.4 ml ATCC medium with 2.5 mM tryptophan and grown to log phase (OD_600_ of 0.5 to 0.7). Cells were harvested by centrifugation (10,000 × *g* for 6 min at 25°C), washed twice with ATCC 974 medium, and resuspended in 4.4 ml fresh ATCC 974 medium with or without 2.5 mM tryptophan supplement.

### DNA cloning and site-directed mutagenesis.

The plasmids and primers used in this study are summarized in Table S1 in the supplemental material. Phusion DNA polymerase was used for high-fidelity PCR cloning, and Taq DNA polymerase was used for colony PCR screening. Site-directed mutagenesis was performed using a QuikChange II XL site-directed mutagenesis kit according to the supplier’s instructions (Agilent Technologies). DNA sequencing was performed by Sanger automated DNA sequencing using an Applied Biosystems model 3130 genetic analyzer (ICBR Genomics Division, University of Florida).

### Immunoblotting.

Cells were harvested by centrifugation (10,000 × *g* for 6 min at 25°C). Cell pellets were suspended in lysis buffer (50 mM Tris-HCl, pH 6.8, 2 M urea, 2% [wt/vol] SDS, and 0.1 mg·ml^−1^ EDTA-free protease inhibitor cocktail [Roche]) with glass beads (Chemglass Life Sciences). Cells were lysed with glass disruptor beads (0.1 mm in diameter) by vigorous vortexing 5 to 7 times (1 min each time with a 1-min incubation on ice in between). Cell debris and glass beads were removed by centrifugation (16,800 × *g* for 20 min at 4°C), and supernatants were transferred into 1.5- ml Eppendorf tubes on ice. The protein concentrations of cell lysates were measured by bicinchoninic acid (BCA) protein assay (Thermo Scientific, Rockville, IL) according to the supplier’s instructions, using bovine serum albumin (BSA; Thermo Scientific) as the protein standard. The cell lysates were mixed with equal volumes of 2× SDS reducing buffer (100 mM Tris-HCl buffer at pH 6.8 with 4% [wt/vol] SDS, 20% [vol/vol] glycerol, 0.6 mg·ml^−1^ bromophenol blue, and 5% [vol/vol] β-mercaptoethanol) and boiled for 10 min. Equivalent protein amounts for each lane, as determined by BCA assay, were separated by SDS-PAGE (12%). Large amounts of cell lysates (around 10 µg) were needed to detect TBP2, which would result in the housekeeping protein being in such a large amount as to be out of the linear dynamic range of immunodetection. Thus, loading controls were done by Coomassie blue staining of parallel gels, which exhibits great linearity in the loading range of 5 to 20 µg of cell lysate (54). Proteins separated by SDS-PAGE (12%) were electroblotted onto polyvinylidene difluoride (PVDF) membranes (Amersham). StrepII-tagged proteins were detected by mouse anti-StrepII polyclonal antibody (Qiagen) followed by goat anti-mouse IgG (whole molecule)–alkaline phosphatase-linked antibody (Sigma). Flag-tagged proteins were detected by alkaline phosphatase-linked anti-Flag M2 monoclonal antibody (Sigma). Immunolabeled proteins on PVDF membranes were visualized by chemiluminescence using CDP-Star (Applied Biosystems) with X-ray film (Hyperfilm; Amersham Biosciences). The intensities of the protein bands were quantified by ImageJ (55).

### 2-D PAGE.

Protein was extracted using a Trizol-based method modified for “salt-loving” proteomes according to protocols described previously (56). Protein samples were further purified from salts by acetone precipitation. Protein pellets were solubilized in 2-D sample buffer (8 M urea, 2 M thiourea, 4% [wt/vol] CHAPS {3-[(3-cholamidopropyl)-dimethylammonio]-1-propanesulfonate}, 0.2% [wt/vol] SDS, and 10 mM Tris-HCl, pH 8.5). Protein concentrations were measured with the EZQ protein quantitation kit according to the manufacturer’s instructions (Thermo Fisher Scientific). Isoelectrofocusing (IEF) was carried out in an IPGphor3 unit (GE Healthcare) on 18-cm immobilized pH gradient (IPG) strips, pH range 3.5 to 4.5, using a cup-loading method to load the sample (GE Healthcare). The following conditions were used for IEF: the voltage was initially set at 300 V for 300 volt hours (Vh) and then ramped up to 1,000 V in 1,000 Vh, ramped up to 10,000 V in 20 kVh, and finally held and focused at 10,000 V for 60 kVh until the current reached steady state at around 20 mA. The strip was mounted onto a 20- by 20-cm 8-to-16% polyacrylamide Tris-glycine gel (Jule Biotechnologies, Inc.) after IEF. Electrophoresis was carried out at 12°C as follows: 10 mA/gel for 1 h and then overnight at a constant current of 12 mA/gel with a limit of 150 V until the dye reached to the bottom of the plate. Immediately after gel electrophoresis, the gel was transferred onto a PDVF membrane (Amersham), followed by immunoblotting.

### Chase assay.

*H. volcanii* cells were grown aerobically in 4 ml ATCC 974 medium to early log phase, subcultured into 5.1 ml fresh ATCC 974 medium, and grown to log phase (OD_600_ of 0.4 to 0.7). Cells were harvested by centrifugation (10,000 × *g* for 6 min at 25°C), suspended in 4.2 ml concentrated salt water stock solution (4.1 M NaCl, 150 mM MgCl_2_·6H_2_O, 140 mM MgSO_4_·7H_2_O, 100 mM KCl, and 20 mM Tris-HCl, pH 7.5), and treated with 20 µg·ml^−1^ actinomycin D (Sigma) and 50 µg·ml^−1^ anisomycin (Sigma). Cells were harvested by centrifugation (10,000 × *g* for 6 min at 4°C) at various intervals (0 to 2 h). Cell pellets were frozen at −80°C and then analyzed by immunoblotting.

### RNA isolation and qRT-PCR.

Total RNA was isolated from *H. volcanii* cells by using an RNeasy minikit according to the supplier’s instructions (Qiagen). DNA was removed by using a Turbo DNA-free kit according to the recommendations of the supplier (Ambion). The level of contaminating DNA after Turbo DNase digestion was below the limit of detection by PCR. The integrity of the RNA was determined by 2.0% (wt/vol) agarose gel electrophoresis. One hundred nanograms of RNA per reaction mixture volume (25 µl) served as the template. One-step quantitative reverse transcriptase PCR (qRT-PCR) was performed using the QuantiTect SYBR green RT-PCR kit following the protocol described in the handbook of the supplier (Qiagen). One-step qRT-PCR was performed under conditions of 50°C for 30 min, 95°C for 15 min, and 40 cycles of 94°C for 15 s, 51°C for 30 s, and 72°C for 30 s, followed by determination of the melting curve by using a CFX96 real-time C1000 thermal cycler (Bio-Rad). A single peak revealed by melting curve analysis indicated a single product. The TBP2 mRNA levels were normalized to the internal standard *ribL*. A standard curve was generated by using a QuantiTect SYBR green PCR kit (Qiagen) following the manufacturer’s protocol. Genomic DNA and purified plasmid encoding Flag-SAMP2 in tandem with TBP2-StrepII served as the templates to test different primer pairs for PCR efficiency. Primers with PCR efficiencies of between 95% and 105% are listed in Table S1 in the supplemental material.

### Enrichment of StrepII-tagged proteins.

For the StrepII pulldown assay, wt and SPS deletion strains carrying plasmid pJAM2201 were grown aerobically in 4 ml ATCC 974 medium to early log phase. Cells were subcultured into 50 ml ATCC 974 medium (in 250-ml baffled flasks at 42°C and 200 rpm) and grown to stationary phase. Cell pellets were harvested, resuspended in lysis buffer (2 M NaCl, 50 mM Tris-HCl, pH 7.4, 1 mg·ml^−1^ EDTA-free protease inhibitor cocktail [Roche]), and lysed by French press 4 to 6 times (24,000 lb/in^2^). Cell debris was removed by centrifugation, and equivalent amounts of cell lysate, as determined by BCA protein assay, were applied to 100 µl Strep-Tactin Superflow resin (Qiagen) equilibrated in Tris buffer (2 M NaCl, 50 mM Tris-HCl, pH 7.4). Nonspecific proteins were removed by washing the Strep-Tactin resin with 40 column volumes of lysis buffer. Bound proteins were eluted from the resin by the addition of 30 µl of 5 mM *d*-desthiobiotin dissolved in Tris buffer. Elution fractions were mixed with equal volumes of 2× SDS reducing buffer, followed by immunoblotting.

For purification of TBP2-StrepII from a large culture, cells were grown to stationary phase in 4 times 1 liter of ATCC 974 medium at 42°C. Cells were resuspended in lysis buffer and lysed by French press as described above. The resulting cell lysate was clarified by centrifugation and filtration (0.45 µm) before being applied to a Strep-Tactin column (1-ml bed volume; GE Healthcare). Unbound proteins were removed by washing the column with 120 ml Tris-salt buffer. TBP2-StrepII proteins were eluted in Tris buffer (2 M NaCl, 50 mM Tris-HCl, pH 7.4) supplemented with 5 mM *d*-desthiobiotin.

### Tandem affinity purification of SAMP2-TBP2.

Wild-type and SPS deletion strains (*ΔubaA* and *Δpan1 Δpan2* strains) expressing plasmid pJAM2201 and wt cells expressing pJAM202c were grown to stationary phase in 2 times 1 liter of ATCC 974 medium at 42°C. After cell lysis, equivalent amounts of total cellular proteins, as determined by BCA protein assay, were passed through a Strep-Tactin column as described above. A total of 3 ml of pooled fractions of eluted proteins were subsequently used for immunoprecipitation by anti-Flag M2 antibody affinity gel according to the supplier’s instructions (Sigma). SAMP2-TBP2 conjugates were eluted in Tris buffer (150 mM NaCl, 50 mM Tris-HCl, pH 7.4) supplemented with 150 ng·µl^−1^ Flag peptide (Sigma) and analyzed by immunoblotting.

### Determining protein mass by ESI-TOF MS.

TBP2 was purified by Strep-Tactin chromatography followed by size exclusion chromatography (Superdex 75; GE Healthcare). Protein purity was determined by Coomassie blue staining of fractions separated by reducing SDS-PAGE. Purified TBP2-StrepII was dialyzed against deionized water 6 times for 2 to 4 h at 4°C by using mini-dialysis tubing (1-kDa molecular-mass cutoff; GE Healthcare). Protein samples were analyzed for accurate mass determination by ESI mass spectrometry. Samples were treated with acetonitrile (50% [vol/vol]) and formic acid (1% [vol/vol]) and immediately loaded into an Agilent 6210 time of flight mass spectrometer (Agilent Technologies, Inc., Santa Clara, CA) with the electrospray ionization source in the positive mode.

### Mapping phosphorylation sites by MS/MS.

TBP2 purified by Strep-Tactin chromatography was separated by 12% SDS-PAGE. Free TBP2 was visualized by staining with Bio-Safe Coomassie (Bio-Rad) and destained in double deionized water. Protein bands were excised from the gel, washed with water, and destained with 50% (vol/vol) acetonitrile in 50 mM ammonium bicarbonate buffer. Proteins in gel were reduced with dithiothreitol (DTT), alkylated with iodoacetamide, and digested with chymotrypsin overnight or subjected to double digestion with endoproteinase Glu-C (*Staphylococcus aureus* protease V8) overnight and then a 4-h incubation with trypsin. Digestions were done at 37°C. Peptides were extracted with a mixture of 70% acetonitrile and 0.1% trifluoroacetic acid and lyophilized.

Phosphopeptides were enriched by using TiO_2_ NuTip microcolumns (GlygenSci, Columbia, MD) according to the method described previously (57). The lyophilized peptides (enriched and flow through) were solubilized in 10 µl of loading buffer (3% [vol/vol] acetonitrile, 0.1% [vol/vol] acetic acid and 0.01% [vol/vol] trifluoroacetic acid) and loaded onto a C_18_ capillary trap cartridge (LC Packings, United States). Peptides were separated on a 15-cm nanoflow analytical C_18_ PepMap column (0.075-mm inner diameter, 3-μm particle size, 100 Å) at a flow rate of 300 nl/min using a nanoLC ultra 1D plus system (AB Sciex, United States). The composition of solvent A was 3% (vol/vol) acetonitrile and 0.1% (vol/vol) acetic acid, whereas solvent B was 97% (vol/vol) acetonitrile and 0.1% (vol/vol) acetic acid. Peptide separation was performed using a linear gradient from 3 to 40% solvent B for 20 min, followed by an increase to 90% solvent B in 5 min and holding for 5 min. The flow was directly sprayed onto an LTQ Orbitrap XL mass spectrometer (Thermo Fisher, Bremen, Germany). MS/MS spectra were acquired in a data-dependent mode. An Orbitrap MS full scan (resolution, 3 × 10^4^; molecular-mass range, 400 to 1,800 Da) was followed by 10 MS/MS scans in the ion trap, which were performed by collision-induced dissociation on the top 10 most abundant ions. The isolation window for ion selection was 3 Da. The normalized collision energy was set at 28%. The dynamic exclusion time was 20 s (58). Additionally, if a phosphate neutral loss of 98, 49, 32.66, or 24.5 *m*/*z* below the precursor ion mass was detected, an additional activation was included in the analysis. This multistage activation (MSA) event was repeated for the top five ions in a data-dependent manner provided the precursor exceeded a threshold of 500 ion counts (59). An inclusion list of the peptides for the TBP2 N terminus was used for the method.

All MS/MS samples were analyzed using Mascot (version 2.4.1; Matrix Science, London, United Kingdom). Mascot was set up to search the database containing *Haloferax volcanii* DS2 proteins and TBP2-StrepII (8087 entries) by assuming the digestion enzyme(s) was (i) chymotrypsin or (ii) Glu-C and trypsin. The Mascot search used a fragment ion mass tolerance of 0.50 Da and a parent ion tolerance of 10.0 ppm. Carbamidomethyl modification of cysteine was specified in Mascot as a fixed modification. Gln to pyro-Glu of the N terminus, deamidation of Asn and Gln, oxidation of Met, and phosphorylation of Ser, Thr, and Tyr were specified in Mascot as variable modifications. Scaffold (version 4.3.4; Proteome Software, Inc., Portland, OR) was used to validate MS/MS-based peptide and protein identifications. Peptide identifications were accepted if they could be established at greater than 90.0% probability by the Scaffold Local false discovery rate (FDR) algorithm. Protein identifications were accepted if they could be established at greater than 99.9% probability and contained at least 1 identified peptide. Protein probabilities were assigned by the Protein Prophet algorithm (60).

## SUPPLEMENTAL MATERIAL

Figure S1  TBP2 abundance is increased by inhibition of Cdc48. TBP2 abundance in wild-type (wt) cells treated with an inhibitor of Cdc48 (14 µM DBeQ, +) or a mock control (ethanol, −) after 24 h. Quantification of relative TBP2 protein levels is indicated below the gels. TBP2 level in mock control was set at 1.0. TBP2 protein levels were determined by anti-StrepII antibody IB. Data were quantified by ImageJ. Equal loading was confirmed by CB staining. See Materials and Methods for details. Download Figure S1, TIF file, 0.4 MB

Figure S2  TBP2 degradation in wild-type strain. Chase assays were performed in wild-type strain expressing Flag-SAMP2 and TBP2-StrepII. Log-phase cells were treated with 20 µg·ml^−1^ actinomycin D and 50 µg·ml^−1^ anisomycin for the indicated times and collected. TBP2 and SAMP2 protein levels were determined by anti-StrepII antibody and anti-Flag antibody, respectively. Equal loading was confirmed by CB staining. Experiments were performed in at least biological duplicates, and representative images are shown. ¥, coexpressed in *trans*. Download Figure S2, TIF file, 0.6 MB

Figure S3  Replacement of Ser2 with Ala or Glu affects TBP2 abundance associated with SPS. (A) TBP2 (wt, S2A, and S2E) protein levels in wild-type (wt) and SPS deletion strains determined by anti-StrepII antibody immunoblotting. TBP2 was C-terminally fused to StrepII, coexpressed with Flag-SAMP2, and monitored by anti-StrepII antibody IB. Equal loading was confirmed by Coomassie blue (CB) staining. Relative levels of TBP2 proteins in wt and SPS deletion strains are indicated below the gels. The level of wt TBP2 protein in the wt strain was set at 1.0. Protein intensities were obtained by ImageJ. Quantification data represent the mean results ± SEM from five independent experiments for the TBP2 S2E variant protein and three independent experiments for the TBP2 S2A variant protein. (B) Histogram showing transcript levels of TBP2 (wt, S2A, and S2E) in wt and SPS mutant strains as revealed by qRT-PCR. The mRNA levels are normalized to the level of the internal standard *ribL*. Data represent one experiment for TBP2 S2A and three experiments for TBP2 S2E. Statistical analysis of TBP2 protein and mRNA intensities relative to those of the wt: *, *P* < 0.05; **, *P* < 0.01 (two-tailed, unpaired Student’s *t* test). ¥, in *trans*. Download Figure S3, TIF file, 0.6 MB

Table S1  List of strains and plasmids used in this study. Table S1, DOCX file, 0.03 MB
