# Erratum for Fu et al., Ubiquitin-Like Proteasome System Represents a Eukaryotic-Like Pathway for Targeted Proteolysis in Archaea

**DOI:** 10.1128/mBio.01192-16

**Published:** 2016-08-09

**Authors:** Xian Fu, Rui Liu, Iona Sanchez, Cecilia Silva-Sanchez, Nathaniel L. Hepowit, Shiyun Cao, Sixue Chen, Julie Maupin-Furlow

**Affiliations:** a Department of Microbiology and Cell Science, Institute of Food and Agricultural Sciences, University of Florida, Gainesville, Florida, USA; b College of Food Science and Technology, Zhongkai University of Agriculture and Engineering, Guangzhou, Guangdong, China; c Proteomics and Mass Spectrometry, Interdisciplinary Center for Biotechnology Research, University of Florida, Gainesville, Florida, USA; d Department of Biology, College of Liberal Arts and Sciences, University of Florida, Gainesville, Florida, USA; e Genetics Institute, University of Florida, Gainesville, Florida, USA

## ERRATUM

Volume 7, no. 3, doi:10.1128/mBio.00379-16, 2016. A researcher’s name cited for unpublished data on PDF page 2 was spelled incorrectly and should appear as “McMillan.”

