# Supplementary figures and images for: Ubiquitin-Like Proteasome System Represents a Eukaryotic-Like Pathway for Targeted Proteolysis in Archaea
Source: mBio. 2016 May 17;7(3):e00379-16. doi: 10.1128/mBio.00379-16 (PMC4895103; doi:10.1128/mBio.00379-16)

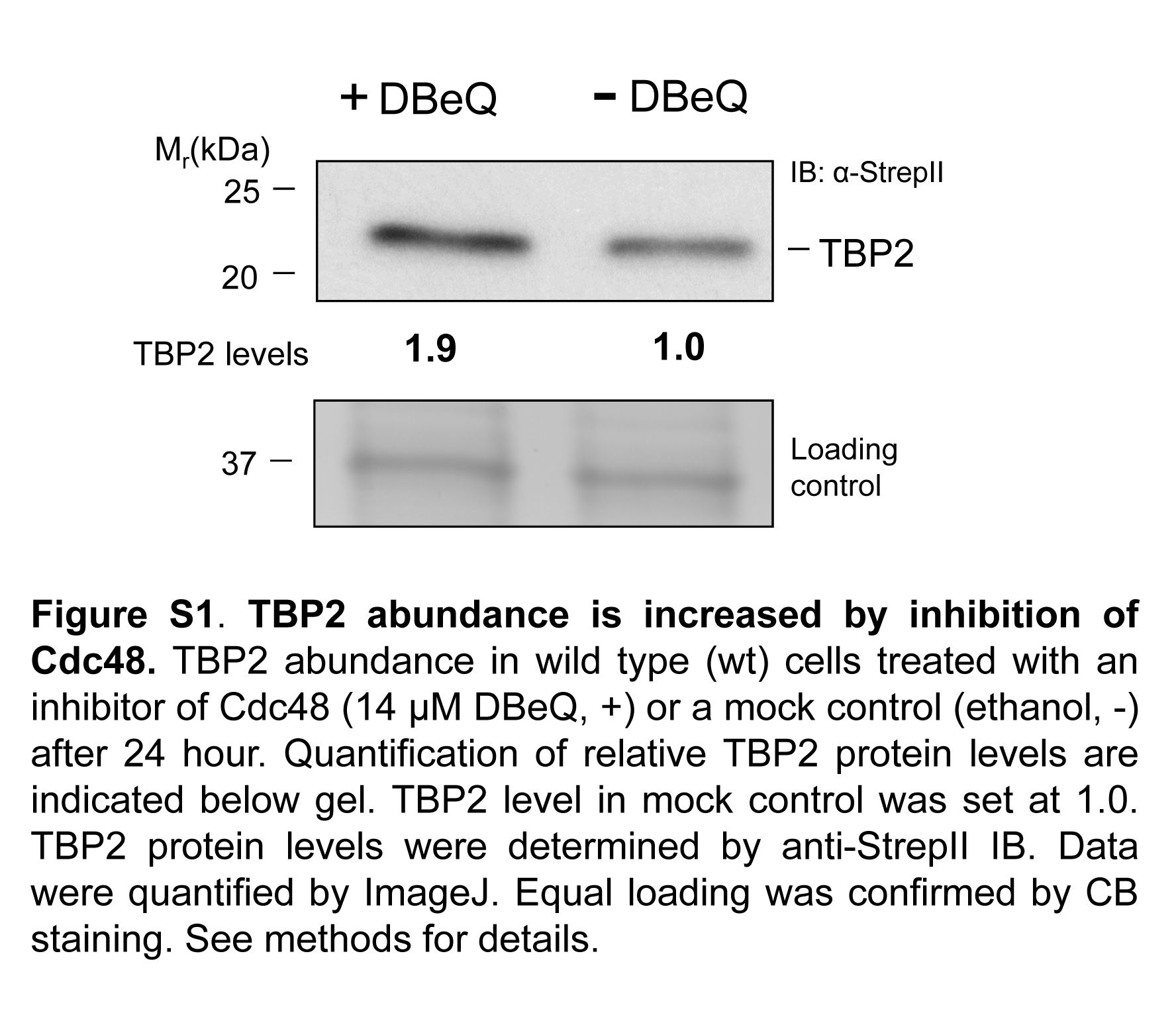

Supplement: Figure S1 — TBP2 abundance is increased by inhibition of Cdc48. TBP2 abundance in wild-type (wt) cells treated with an inhibitor of Cdc48 (14 µM DBeQ, +) or a mock control (ethanol, −) after 24 h. Quantification of relative TBP2 protein levels is indicated below the gels. TBP2 level in mock control was set at 1.0. TBP2 protein levels were determined by anti-StrepII antibody IB. Data were quantified by ImageJ. Equal loading was confirmed by CB staining. See Materials and Methods for details. Download [file mbo002162815sf1.tif]

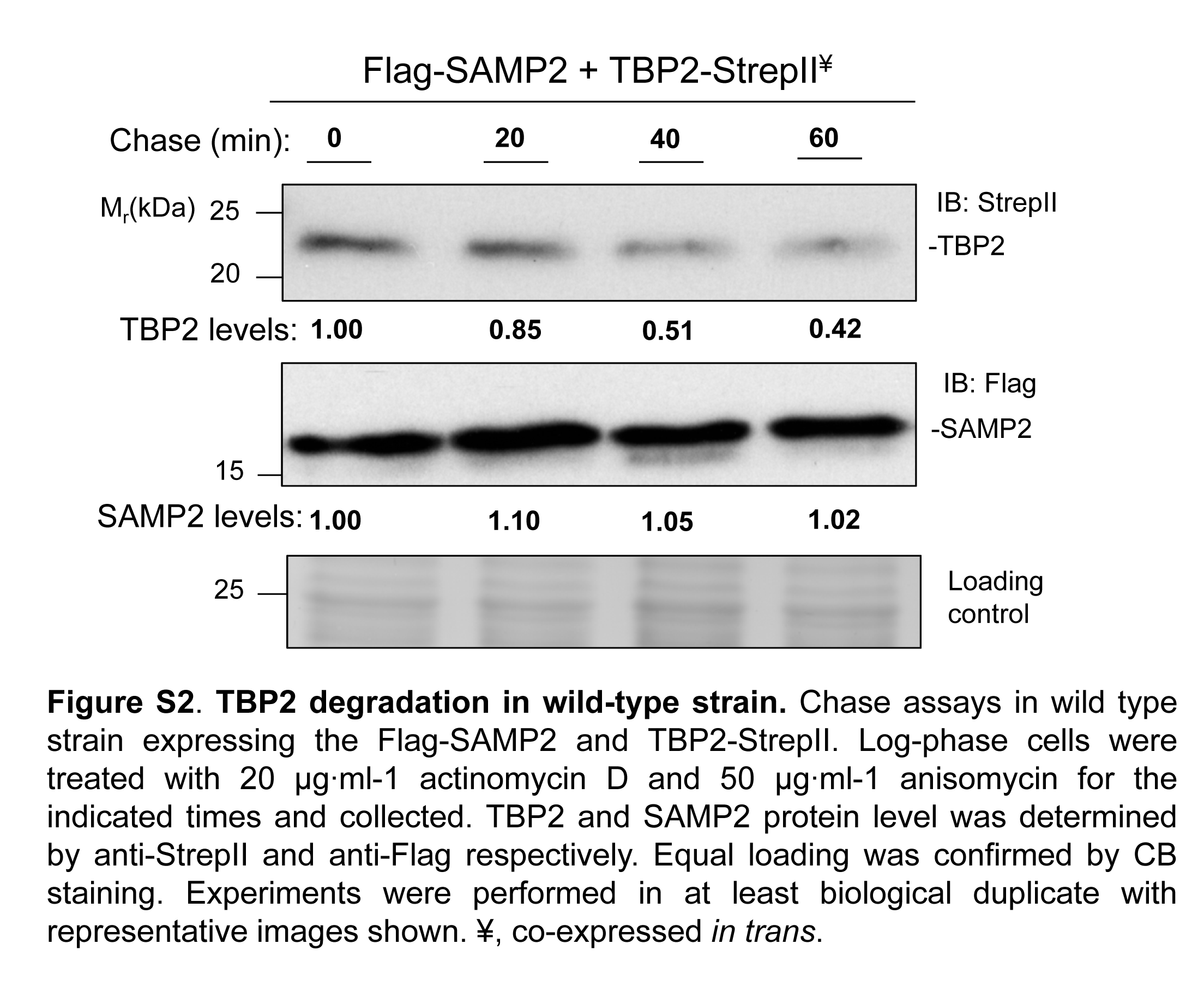

Supplement: Figure S2 — TBP2 degradation in wild-type strain. Chase assays were performed in wild-type strain expressing Flag-SAMP2 and TBP2-StrepII. Log-phase cells were treated with 20 µg·ml−1 actinomycin D and 50 µg·ml−1 anisomycin for the indicated times and collected. TBP2 and SAMP2 protein levels were determined by anti-StrepII antibody and anti-Flag antibody, respectively. Equal loading was confirmed by CB staining. Experiments were performed in at least biological duplicates, and representative images are shown. ¥, coexpressed in trans. Download [file mbo002162815sf2.tif]

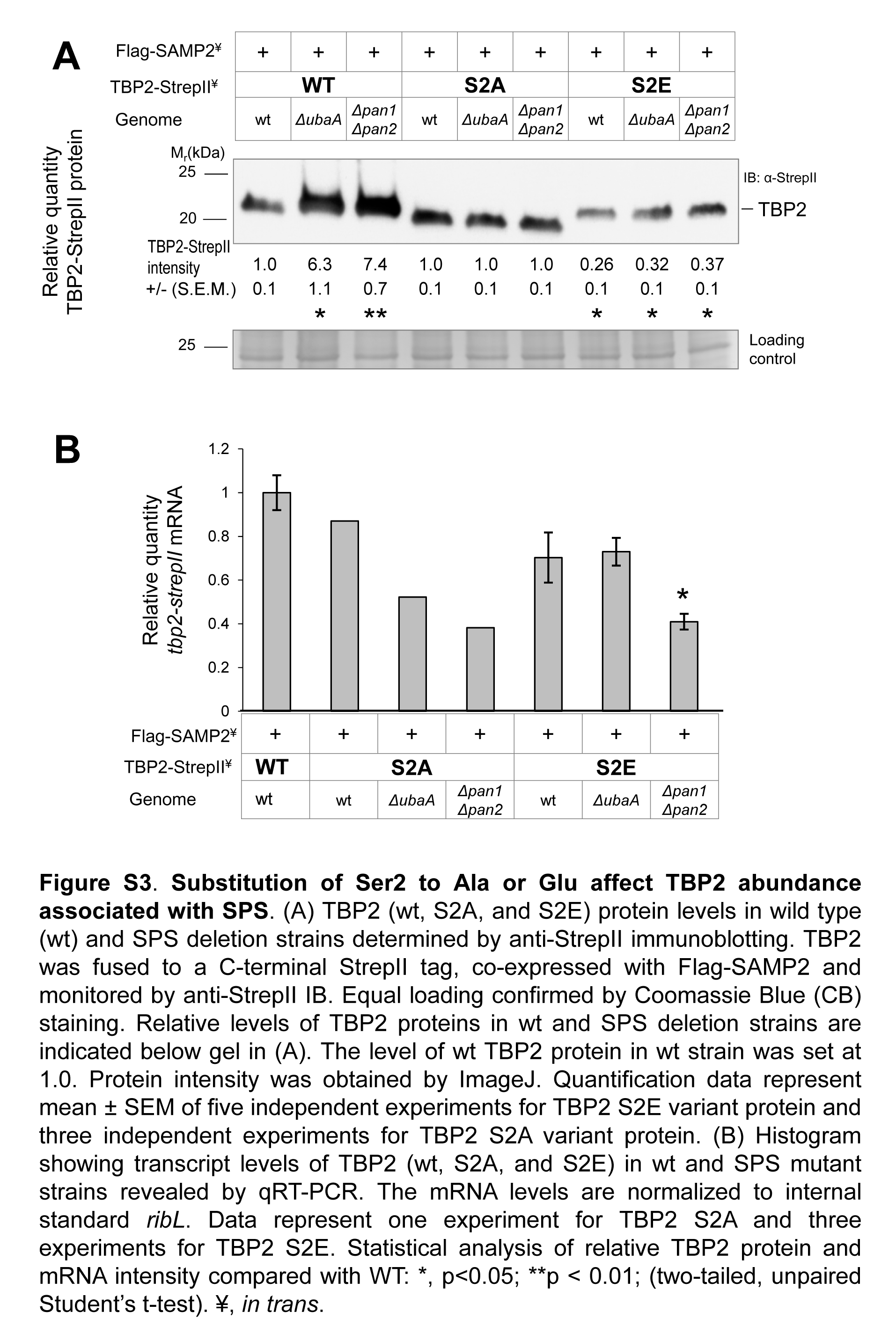

Supplement: Figure S3 — Replacement of Ser2 with Ala or Glu affects TBP2 abundance associated with SPS. (A) TBP2 (wt, S2A, and S2E) protein levels in wild-type (wt) and SPS deletion strains determined by anti-StrepII antibody immunoblotting. TBP2 was C-terminally fused to StrepII, coexpressed with Flag-SAMP2, and monitored by anti-StrepII antibody IB. Equal loading was confirmed by Coomassie blue (CB) staining. Relative levels of TBP2 proteins in wt and SPS deletion strains are indicated below the gels. The level of wt TBP2 protein in the wt strain was set at 1.0. Protein intensities were obtained by ImageJ. Quantification data represent the mean results ± SEM from five independent experiments for the TBP2 S2E variant protein and three independent experiments for the TBP2 S2A variant protein. (B) Histogram showing transcript levels of TBP2 (wt, S2A, and S2E) in wt and SPS mutant strains as revealed by qRT-PCR. The mRNA levels are normalized to the level of the internal standard ribL. Data represent one experiment for TBP2 S2A and three experiments for TBP2 S2E. Statistical analysis of TBP2 protein and mRNA intensities relative to those of the wt: *, P < 0.05; **, P < 0.01 (two-tailed, unpaired Student’s t test). ¥, in trans. Download [file mbo002162815sf3.tif]
