# Supplementary material for: Ubiquitin-Like Proteasome System Represents a Eukaryotic-Like Pathway for Targeted Proteolysis in Archaea
Source: mBio. 2016 May 17;7(3):e00379-16. doi: 10.1128/mBio.00379-16 (PMC4895103; doi:10.1128/mBio.00379-16)
Supplement: Table S1 — List of strains and plasmids used in this study. [file mbo002162815st1.docx]

**Table S1.** List of strains, plasmids and used in this study.

| **Strain, plasmid** | **Description^a^** | **Source or reference** |
| --- | --- | --- |
| **Strain:** |  |  |
| ***E. coli*** |  |  |
| Top10 | F^–^ *recA1 endA1 hsdR17*(r_K_^–^ m_K_^+^) *supE44 thi-1 gyrA relA1* | Invitrogen |
| GM2163 | F^–^ *ara-14 leuB6 fhuA31 lacY1 tsx78 glnV44 galK2 galT22 mcrA dcm-6 hisG4 rfbD1 rpsL136 dam13*::Tn*9 xylA5 mtl-1 thi-1 mcrB1 hsdR2* | New England Biolabs |
| XL10-Gold | Tet^r^ *Δ(mcrA)183 Δ(mcrCB-hsdSMR-mrr)173 endA1 supE44 thi-1 recA1 gyrA96 relA1 lac* Hte [F´ *proAB lacIqZΔM15 Tn10* (Tet^r^) Amy Cm^r^] | Agilent Technologies |
| ***Hfx. volcanii*** |  |  |
| H26 | DS70 *ΔpyrE2* | (1) |
| HM1052 | H26 *ΔubaA* | (2) |
| SC100 | H26 *Δjamm1* | (3) |
| SC102 | H26 *Δjamm2* | This study |
| SC115 | H26 *Δjamm1Δjamm2* | This study |
| GZ109 | H26 *Δpan1* | (4) |
| GZ108 | H26 *Δpan2* | (4) |
| GZ132 | H26 *Δpan1Δpan2* | (4) |
| GZ138 | H26 P*_tnaA_-psmB* | (4) |
| NN2 | H26 *Δcdc48b* | This study |
| NN3 | H26 *Δcdc48c* | This study |
| **Plasmid:** |  |  |
| pJAM202c | Amp^r^; Nov^r^; *Hfx. volcanii*-*E. coli* shuttle plasmid, empty vector | (5) |
| pJAM2202 | Amp^r^; Nov^r^; pJAM202c carries P2*_rrn_*-TBP2-StrepII | This study |
| pJAM2201 | Amp^r^; Nov^r^; pJAM202c carries P2*_rrn_*-Flag*-*SAMP2, TBP2-StrepII | This study |
| pJAM2230 | Amp^r^; Nov^r^; pJAM202c carries P2*_rrn_*-Flag*-*SAMP2, TBP2-StrepII S2A | This study |
| pJAM2236 | Amp^r^; Nov^r^; pJAM202c carries P2*_rrn_*-Flag*-*SAMP2, TBP2-StrepII S2E | This study |
| pJAM2265 | Amp^r^; Nov^r^; pJAM202c carries P2*_rrn_*-Flag*-*SAMP2, TBP2-StrepII S2L | This study |

| **Primer Pair** | **Primer sequence (5’-3’)^b^** | **Description** |
| --- | --- | --- |
| HVO_1727 NdeI fwd  HVO_1727 KpnI rev | 5’-CGTCACCATATGAGTGGGCCGGCAGAC-3’  5'-CTTGGACTGCTCGGCGGTACCCC -3' | TBP2-StrepII |
| HVO_1727 S2A inv fwd  HVO_1727 S2A inv rev | 5’-GGAGATATACATATGGCCGGGCCGGCAGACTCC-3’  5'-GGAGTCTGCCGGCCCGGCCATATGTATATCTCC-3' | TBP2-StrepII S2A |
| HVO_1727 S2E inv fwd  HVO_1727 S2E inv rev | 5’-GGAGATATACATATGGAGGGGCCGGCAGACTCC-3’  5'-GGAGTCTGCCGGCCCCTCCATATGTATATCTCC-3' | TBP2-StrepII S2E |
| HVO_1727 S2L inv fwd  HVO_1727 S2L inv rev | 5’-GGAGATATACATATGCTCGGGCCGGCAGACTCC-3’  5'-GGAGTCTGCCGGCCCGAGCATATGTATATCTCC-3' | TBP2-StrepII S2L |
| TBP2-StrepII qRT-PCR fwd  TBP2-StrepII qRT-PCR rev | 5'-CGGGTAGCTGAGATCCTAGAAA-3'  5'-CGATGCGACTACGTTCTGAAT-3' | 104-bp probe for mRNA encoding TBP2-StrepII |
| HVO_0484 qRT-PCR fwd  HVO_0484 qRT-PCR rev | 5'-GCGAGTACATCACGGGTATC-3'  5'-CACTTCCTCTTCGACCTTCAG-3' | 116-bp probe for *ribL* (internal standard) |

^a^Amp^r^, ampicillin resistance; Nov^r^, novobiocin resistance; Tet^r^, tetracycline resistance; Cm^r^, chloramphenicol resistance.

^b^Restriction enzyme or site-directed mutagenesis sites are underlined.

**Supplemental References**

1. **Allers T, Ngo HP, Mevarech M, Lloyd RG.** 2004. Development of additional selectable markers for the halophilic archaeon *Haloferax volcanii* based on the *leuB* and *trpA* genes. Appl Environ Microbiol **70:**943-953. <http://dx.doi.org/10.1128/AEM.70.2.943-953.2004>

2. **Miranda H, Nembhard N, Su D, Hepowit N, Krause D, Pritz J, Phillips C, Söll D, Maupin-Furlow J.** 2011. E1- and ubiquitin-like proteins provide a direct link between protein conjugation and sulfur transfer in archaea. Proc Natl Acad Sci U S A **108:**4417-4422. <http://dx.doi.org/10.1073/pnas.1018151108>

3. **Cao S, Hepowit N, Maupin-Furlow J.** 2015. Ubiquitin-like protein SAMP1 and JAMM/MPN+ metalloprotease HvJAMM1 constitute a system for reversible regulation of metabolic enzyme activity in Archaea. PLOS ONE **10:**e0128399. <http://dx.doi.org/10.1371/journal.pone.0128399>

4. **Zhou G, Kowalczyk D, Humbard M, Rohatgi S, Maupin-Furlow J.** 2008. Proteasomal components required for cell growth and stress responses in the haloarchaeon *Haloferax volcanii*. J Bacteriol **190:**8096-8105. <http://dx.doi.org/10.1128/JB.01180-08>

5. **Reuter C, Uthandi S, Puentes J, Maupin-Furlow J.** 2010. Hydrophobic carboxy-terminal residues dramatically reduce protein levels in the haloarchaeon *Haloferax volcanii*. Microbiology-SGM**:**248-255. <http://dx.doi.org/10.1099/mic.0.032995-0>
